# Natural Occurrence of Deoxynivalenol in Cereal-Based Baby Foods for Infants from Western Poland

**DOI:** 10.3390/toxins13110777

**Published:** 2021-11-04

**Authors:** Kinga Mruczyk, Angelika Cisek-Woźniak, Małgorzata Mizgier, Rafał W. Wójciak

**Affiliations:** 1 Department of Dietetics, Faculty of Physical Culture in Gorzów Wlkp., Poznan University of Physical Education, Estkowskiego 13, 66-400 Gorzów Wielkopolski, Poland; a.cisek@awf-gorzow.edu.pl (A.C.-W.); m.mizgier@awf-gorzow.edu.pl (M.M.); 2 Department of Clinical Psychology, Poznan University of Medical Science, 60-812 Poznań, Poland; rafwoj@ump.edu.pl

**Keywords:** deoxynivalenol, mycotoxin, occurrence, cereal-based baby foods, Poland

## Abstract

The study examined 110 samples of baby products based on rice, wheat, maize and multi-grains available on the western Polish market in order to detect the level of deoxynivalenol (DON) by means of HPLC (high-performance liquid chromatography) with a fluorescence detector (HPLC-FLD). DON was detected in 9.09% of the infant food samples, with an average and maximum level of 107.8 ± 30 and 148 μg/kg, respectively. The highest concentration of DON was detected in food for infants: wheat-based (mean 121 ± 7.07, 4.8%), multi-grain (mean 118 ± 5.65, 4.25%) and maize-based (mean 100 ± 37.96; 35.30%). No high DON content and high estimated daily intake were observed in the analyzed products. However, in order to minimize the harmfulness associated with the presence of DON in food for infants and young children, a risk assessment should be performed based on the monitoring results.

## 1. Introduction

Nowadays, cereals are the most important source of energy in the world. In developed countries, about 30% of daily calories come from cereals, while in developing and poor countries they account for more than 60% and 80%, respectively [1]. Cereal-based foods contribute significantly to human exposure to mold metabolites and mycotoxins. These can cause numerous adverse symptoms, both acute and chronic [2,3].

Mycotoxins are secondary metabolites produced by a wide variety of fungal species and pose a significant risk to the food chain [4]. The Food and Agriculture Organization (FAO) has determined that exposure to mycotoxins through the use of the world’s food crops is close to 25% [5]. Mycotoxins can be produced under the influence of many environmental factors. These factors include temperature, insect damage, water activity, inadequate storage conditions and drought. Among the mycotoxins, one of the most important fungal toxins posing a risk to food is DON.

*Fusarium graminearum* and *F. culmorum* are responsible for the production of deoxynivalenol (DON) and can also be the cause of FHB (Fusarium Head Blight) cereal diseases, particularly in wheat, barley, oats and maize [6]. DON is not classified by the IARC (International Agency for Research on Cancer) as carcinogenic, but studies in different animal species have shown its teratogenicity, genotoxicity, cytotoxicity and immunotoxicity [4,7].

DON can be found in many agricultural products. It is usually produced before harvesting in the field. DON is a very stable mycotoxin, resistant to industrial processing and therefore may occur in products such as processed cereal-based foods for infants and children.

The EC (European Commission) categorizes processed cereal baby foods as foods intended for infants (<12 months) and young children (1 to 3 years of age) as an addition to a diet or as a gradual modification of the diet to adapt to ordinary food [8]. Many studies have shown that mycotoxin contamination is also a problem in these foods [9,10,11]. The increased susceptibility to contamination in this population group has forced the European Commission to set maximum levels of DON and other mycotoxins in foods intended for infants and young children at 200 μg/kg. This value is more than half that of the maximum levels of DON in cereals and other products and more than eight times lower than the maximum levels of DON in unprocessed wheat, oats and maize permitted for human consumption [12]. Studies show that DON is the least lethal of the trichothecene mycotoxins, but, in children, a reaction occurs a few hours after the ingestion of DON [4].

There is a need for babies aged 4–6 months to gradually replace their first food, which is mother’s milk, with cereal products [13]. Initially, infants are given gluten-free cereals made of rice and maize, and then, gradually, multi-grain products. As a consequence, exposure to DON seems inevitable, as there are many sensitive products in cereal-based baby food.

In newborns, the biotransformation capacity of xenobiotics is slower than in adults, resulting in a greater circulation of chemicals absorbed from the food [14]. Consequently, because of their poorly developed detoxification system, lower body weight and higher metabolic rates, infants are more vulnerable to the side effects of mycotoxins compared to adults [15,16].

Most foods for infants and young children are grain-based, which increases the likelihood of many mycotoxins. Therefore, due to the limited availability of data in Europe and Poland on the presence of mycotoxins in food for infants and young children, research in this area is necessary. In the last decade, several studies have been conducted on the contamination of infant foods by mycotoxins [17,18,19,20,21].

Therefore, the aims of this study were: to determine the presence and levels of DON in cereal products for infants available on the Polish market, to compare this with the maximum levels specified by the EU and to estimate the daily consumption of DON in infants of different ages.

## 2. Results

### 2.1. The Presence of DON in Cereal-Based Baby Foods

The presence of DON was found in two of the three brands of infant cereal (Table 1). Regarding DON, samples containing mycotoxin accounted for 10 out of 110 (9.09%) (Table 2). Of the brands studied, only one (brand C) of them (*n* = 5) showed no DON contamination in the products. DON was detected in 15% of samples (brand A) with maximum and average levels of 148 and 104.6 ± 37.968 μg/kg, respectively, in all types of infant food. The DON levels in 100% of A and B brand samples were lower than allowed by the EC [13]. For brand B, 6% of samples were contaminated with DON, and the mean level of DON in the positive samples was 121.0 ± 5.507 μg/kg.

Table 2 shows that the DON content ranges from 62 to 148 μg/kg, while the average values are 107.8 μg/kg. The highest content of the tested mycotoxin, 148 μg/kg, was detected in the sample containing maize as the main component. The maximum permissible level of DON (200 μg/kg) was not exceeded, and all the values of the 110 tested food samples were below this value.

In our study, six samples of cereal-based products for infants under six months of age contained the mycotoxin analyzed. These were maize-containing products (6). Two samples of cereal products for children over nine months of age were positive for mycotoxin; the samples contained wheat (2). In multi-grain products intended for children over 12 months of age, only two samples contained DON.

The test samples were also compared in terms of the DON content in the products: gluten-free (*n* = 35) and containing gluten (*n* = 75). Grain-based cereals that did not contain gluten, intended mainly for infants up to 6 months of age, contained maize and rice, while products containing gluten included wheat, rye, barley and spelt. No statistically significant differences were found for DON (*p* > 0.05) between the two types of samples.

### 2.2. Estimation of Daily Intakes for DON

The study also estimated the daily intake of DON from cereal products in three age groups (6, 9 and 12 months). Estimated Daily Intake (EDI) values are calculated by the formula:

EDI = K (g/day) × Cm (μg/kg)/bw (kg)

where EDI is the estimated daily intake for DON (μg/kg bw/day); K is the cereal intake (g/day) for each age group; Cm is the mean concentration of mycotoxins (μg/kg); and bw (kg) is the body weight at different ages of infancy.

In Poland, the intake of cereals in the tested age groups was 7.4 g per day for infants aged 6 months, 16 g per day for children aged 9 months and 24 g per day for infants aged 12 months [22].

The mean DON contamination values of cereal-based products for infants of different ages were used from Table 3.

The average body weight of the study group was determined based on the WHO guidelines (WHO Child Growth Standards) [23]. These values were determined at the level of 7.4 kg for infants aged 6 months, 8.9 kg for infants aged 9 months and 9.2 kg for infants aged 12 months.

DON has been classified into group 3 by the IARC, which is not carcinogenic to humans [24]. The Tolerable Daily Intake (TDI) for DON was determined by EFSA at 1 μg DON/kg body weight/day [25]. These intakes were 10%, 23% and 31% of the TDI established by the EFSA (European Food Safety Authority), respectively (Table 4).

To estimate infant exposure to dietary deoxynivalenol intake expressed as ug/kg bw/day, the body weight specific to infant age was calculated based on the percentile chart of body weight of Polish infants. Infant body weight was assumed at the level of the 50th percentile [23].

## 3. Discussion

JECFA (the Joint FAO/WHO Expert Committee on Food Additives) recognized food contamination by mycotoxins as a serious threat to public health [26]. In the System/Food Monitoring Evaluation Program (GEMS/Food), mycotoxins were identified as priority food contaminants by the WHO [27]. These compounds are often thermostable and are not usually removed during cooking and sterilization [28].

Diet may be one of the reasons why humans are exposed to mycotoxic contamination. It may contain natural products or synthetic chemicals that may pose a toxic risk to the consumer, including the young.

Research on DON contamination in cereal-based infant foods is quite limited. Table 5 shows the studies on the presence of DON in baby food. According to EFSA, maize, barley, wheat and oats are the most susceptible to Fusarium infestation and the accumulation of DON in the final product [29].

In our study, out of the 110 samples tested, only 9.1% of the samples contained DON. The prevalence of DON is lower than that found in Spain by Rubert et al. [30]. Higher contamination values of 76% were also reported by Juan et al. [9], 65% by Sartori et al. [31], 57% by Zhang et al. [32] and 44% of contaminated DON samples were shown by Pereira et al. [10].

EFSA published a scientific opinion related to the presence of DON in food and feed. That opinion indicated that data on the prevalence of DON in ‘baby food for infants and young children’ are especially rare. It was also indicated that some of the scientific data contained limited information, which at times made it difficult to interpret [25].

Only 10 analyzed samples contained DON and it was below the maximum value. Cereals in which DON has been detected are maize-based products (6), rice-based products (2) and wheat-based products (2).

For comparison, in the study by Juan et al. [9], DON was detected in 19 of the 25 samples tested, and the products contained wheat, rice and maize as the main components of contaminated DON samples.

In this study, it was observed that there is a higher frequency of contamination and a higher level of DON in maize- and wheat-based products, but not exceeding half of the maximum limit of 200 μg/kg. The findings were similar in studies from Italy; the mean DON was 103 μg/kg in maize- and wheat-based products [9], while in Spain, the mean DON was 131 μg/kg in cereal-based baby food [33].

Despite the strict regulations introduced in 2006, many studies have found that DON contamination in products exceeds maximum limits [32,33].

The most frequently contaminated products that exceed the permitted standards are multi-grain cereal products for infants and young children. Moreover, in those where barley, wheat or maize was the dominant grain in the recipe, the highest maximum levels were observed [9,34]. There are also studies in which no DON contamination was found in 57 analyzed samples of baby food from the Spanish market [35].

There were also studies in which, as in ours, insignificant amounts of DON were detected [9,36]. Studies by many authors indicate the heterogeneity of DON contamination in samples for infants and young children, which justifies the need for a more in-depth study of DON contamination and its metabolites in cereals and cereal products for infants and young children.

In our study, the EDI of DON through the consumption of cereals for infants at 6, 9 and 12 months was 0.10, 0.23 and 0.31 µg/kg body weight/day, respectively. In Spain, the intake of DON with infant cereal products was found to be 0.08 μg/kg body weight/day, which was lower than in our study [37].

## 4. Conclusions

The results of this study provide information related to assessing the occurrence of and exposure to DON in cereal-based baby food. Ten percent of the baby cereal samples were contaminated with DON. No sample exceeded the EU maximum level for DON.

Infants and young children are a very vulnerable group and are particularly susceptible to the harmful effects of DON through a restricted diet based on various types of cereals. The study estimated the DON consumption of cereals for infants at 6, 9 and 12 months to be 10, 23 and 31% of the TDI set by EFSA at 1 μg/kg body weight/day, respectively. From a toxicological point of view, it is imperative to keep the contamination by DON at minimum levels.

Our results emphasize the need for producers to apply all possible preventive measures. One such method is HACCP (Hazard Analysis and Critical Control Points), a procedure for reducing and preventing the contamination of raw materials used in the production of food for infants and young children. These measures should ensure coordinated surveillance programs to monitor maximum levels in processed cereal-based foods and baby foods for infants and young children. In addition, a Good Agricultural Practice (GMP) commitment is necessary to eliminate DON from products. In addition, optimizing the storage and transportation conditions of cereals can be an effective approach to inhibiting the formation of DON-producing fungi.

In the future, it is planned to extend the scope of research on mycotoxins in products for young children from Poland.

## 5. Materials and Methods

### 5.1. Samples Collection

A total of 110 samples of infant cereals were randomly collected from different supermarkets, pharmacies and retailers in the west of Poland during 2017 and 2018. The samples came from 3 brands, which account for the majority share of products for infants on the Polish market.

In our study, the samples were divided into three groups, depending on the age of the infants for whom they were intended: products for infants aged 6 months (*n* = 35), 9 months (*n* = 48) and 12 months (*n* = 27).

According to the manufacturers’ declarations on the labels, products for infants up to 6 months of age contained mainly one or two grains—maize and rice—while the multi-grain products intended for infants aged 9 and 12 months also contained wheat, barley, oats, rye, maize and rice.

The sampling and preparation of the samples was carried out in accordance with Commission Regulation 401/2006 [38] which lays down the methods of sampling for the official control of the levels of mycotoxins in foodstuffs.

Aggregate samples weighing not less than 1 kg were collected by pooling the three incremental samples, with the minimum weight of the sample being 500 g.

The samples were delivered to the laboratory within 48 h, and during transport, the samples were stored in a dry and cool place.

The prepared portions intended for testing were ground into a fine powder with a thickness of 1.0 mm using an analytical grinder. Until the analysis was performed, the samples were stored at −4 °C.

Mycotoxin was isolated from products using a R-Biopharm Rhône’s Donprep^®^ immunoaffinity column for mycotoxin extraction, according to the manufacturer’s procedures. Mycotoxin were analyzed by high-performance liquid chromatography with fluorescence detection (HPLC-FLD).

### 5.2. Mycotoxin Analysis

To determine DON, the method used was the National Institute of Hygiene Methodology—“Determination of Fusarium toxins—deoxynivalenol in cereals and its products by high-performance liquid chromatography with purification using immunoaffinity columns”.

#### 5.2.1. Basic DON Solutions

From the certified standard solution of acetonitrile with a DON concentration of 100 µg/mL, 500 ug was pipetted into a 5 mL volumetric flask, evaporated and then methanol HPLC was added up to the mark. From the resulting stock solution of 10 ug/mL, the volumes given in the table were pipetted into a 2 mL volumetric flask and made up to the mark with 9.5% methanol for HPLC (Table 6).

#### 5.2.2. Apparatus and Materials

Laboratory balance, laboratory shaker, homogenizer, laboratory centrifuge 4000 rpm, kit for evaporating the nitrogen stream with a water bath at 40 °C, 0.45 µm membrane filter, Whatman glass fiber filter retaining particles with a diameter of 1.6 um or less, SPE (Solid Phase Extraction) kit with a vacuum pump, stand and 75 mL trays, 100 and 250 mL volumetric flasks and automatic pipettes.

The HPLC kit comprised: a gradient pump providing a flow rate of 1.0 mL/min, a 100 µL dosing system, a C18-type RP-HPLC column, 250 mm × 4.6 mm, providing separation to baseline of the DON peak from all other peaks (the peak overlap was no greater than 10%), a C18 Column 20 mm (Waters), UV-VIS detector and integration and data acquisition system.

#### 5.2.3. Determination Method

Approximately 20 g of the test sample was weighed into a beaker to the nearest 0.1 g. To this, 4 g PEG (polyethylene glycol) and 80 mL water were added. This was then homogenized at a high speed for 3 min and centrifuged at 4000 rpm for 15 min. The extract was filtered through a glass fiber filter. Then, 2.0 mL of the clear filtrate was pipetted onto an Immunoaffinity column (IAC).

#### 5.2.4. Purification Using the IAC

The filtrate was passed through the IAC at a flow rate of about 1 mL/min. The column was washed with about 5 mL of water at a flow rate of 1 mL/min and dried by passing air through the column using a syringe for 10 s. DON was eluted by applying 2 mL of methanol HPLC to the column and was then passed through using gravity. The eluate was collected in a test tube. Any residual solvent was removed from the column by passing air through the column.

#### 5.2.5. HPLC Analysis

Operating conditions: mobile phase flow rate—1.00 mL/min, volume supplied 100 uL, column temperature 40 °C, autosampler temperature 4 °C and operating wavelength 220 nm. A standard curve was prepared from the standard solutions described above under the same conditions as were used during the analysis. The curve is plotted as the DON peak area–standard concentration.

#### 5.2.6. Verification of Research Method

The limit of detection (LOD) was 0.009 ug/mL, and the limit of quantification (LOQ) was 0.019 ug/mL. The linear range of the calibration curve was 0.20–2.00 ug/mL. The linearity was r = 0.9997, the sensitivity of the method 42.152, the correctness of the method 12.93%, the precision of the method 1.59% and the recovery was 83%. The composite standard uncertainty was 7% and the expanded measurement uncertainty was 13%. The recovery values for DON were appropriate and in line with the recovery values specified in the requirements of Commission Regulation (EC) No. 401/2006 [38], that is, between 60% and 110% for DON.

The DON content of the sample is expressed in μg/kg calculated as:

$$C=\frac{c\times V3\times V2}{m\times V1}\times 1000$$

where:

*C*—concentration of DON in the product (µg/kg)
*c*—concentration read from the calibration curve (µg/mL)
*V*1—volume applied to the IAC column (2 mL)
*V*2—final volume eluted from IAC (0.5 mL)
*V*3—volume of extraction solvent (80 mL)
*m*—sample weight (20 g)

### 5.3. Statistical Analysis

The results of the research were analyzed using the STATISTICA v.13.3 statistical program. Differences in mycotoxin contamination in the groups were tested using the Mann–Whitney test. Correlations at the level of significance α not exceeding 0.05 (*p* < 0.05) were considered statistically significant.

## Figures and Tables

**Table 1:** DON content (µg/kg) in different brands of cereal-based baby food.

Brand	Total Samples	Positive Samples		Concentration		SD
		N	%	Mean	Range Min.–Max.	
A	39	6	15	104.6	62–148	37.968
B	66	4	6	121.0	116–126	5.507
C	5	0	0	-	-	-

**Table 2:** Occurrence of DON in cereal-based products.

Product	Total Samples	Positive Samples		Concentration		SD
		N	%	Mean (µg/kg)	Range Min.–Max.	
Generally	110	10	9.09	107.8	62–148	30,205
Mix	47	2	4.25	118	114–122	5656
Maize	17	6	35.30	100	62–148	37,968
Wheat	42	2	4.80	121	116–126	7071
Rise	4	0	-	-	-	-

**Table 3:** Occurrence of DON in cereal-based products by age.

Age	Total Samples	Positive Samples		Concentration		SD
		N	%	Mean (µg/kg)	Range Min.–Max.	
6	35	6	17.1	104.7	62–148	37.968
9	48	2	4.2	121.0	116–126	7.071
12	27	2	7.4	118.0	114–122	5.656

**Table 4:** Sample data on the consumption of food produced with cereals for infants and young children (*n* = 110) by age group.

	Mean Consumption (g/Day)	Mean Concentration (µg/kg)	Exposure μg/kg bw/Day	% of Exposure	Tolerable Daily Intake μg/kg bw/Day
6 months	7.4	104.7	0.10	10	1.0
9 months	16.0	121	0.23	23	
12 months	24.0	118	0.31	31	

**Table 5:** Occurrence of DON in cereal-based baby food products in different countries.

Country	Samples Analyzed (N)	Positive Samples		Concentration		Reference
		N	%	Range	Mean	
Italy	75	19	25	Ld-268	102.60	Juan et al., 2014
Spain	35	9	26	70–210	Nd	Rubert at al., 2012
	30	12	-	Max: 286	131	Cano Sancho et al., 2011
Portugal	9	4	-	29–271	160.60	Pereira et al., 2015
United States	64	42	66	Max: 146.50	Nd	Al.-Taher et al., 2017
	147	96	65	34–258	Nd	Zhang et al., 2014
Tunisia	32	20	63	10–110	30	Queslati et al., 2017

Nd—Not defined. Ld—Limit of detection. Max—Maximum level.

**Table 6:** Preparing the standard solution.

Standard Solution	10 ug/mL Extracted from the Basic Solution	Mass Concentration of DON ug/mL
1.	40	0.2
2.	100	0.5
3.	150	0.75
4.	200	1
5.	400	2

These certified solutions were used to prepare the fortification solution.
